# Patient preferences for emergency or planned hip fracture surgery: a cross-sectional study

**DOI:** 10.1186/s13018-016-0454-2

**Published:** 2016-10-17

**Authors:** Abhinav Aggarwal, Ian A. Harris, Justine M. Naylor

**Affiliations:** 1 Orthopaedic Department, Liverpool Hospital, Liverpool, 2170 New South Wales Australia; 2 South West Sydney Clinical School, University of New South Wales, Liverpool, 2170 New South Wales Australia; 3 Whitlam Orthopaedic Research Centre, Ingham Institute for Applied Medical Research, Level 2, 1 Campbell St, Liverpool, 2170 New South Wales Australia

**Keywords:** Patient preferences, Orthopaedic surgery, Hip fracture

## Abstract

**Background:**

The ideal timing of surgical management for hip fractures remains controversial. Currently, individual surgeon preference and departmental resources guide decision making regarding the use of emergency or planned operating lists for hip fracture surgery. We evaluated patient preference for emergency or planned surgery.

**Methods:**

102 patients awaiting surgery for a hip fracture at a tertiary hospital were surveyed in this cross-sectional study. After being informed of the benefits and risks associated with an emergency or planned operation, the patients were asked to indicate a hypothetical preference for surgical operating time. They were then asked to give an importance value for six factors that may influence decision making including consultant supervision, operative timeliness, surgical cancellation, after hours operation, length of hospital stay and repeated fasting. For each factor, absolute importance was rated from 0 to 10, and factors were independently ranked for relative importance from 1 to 6. An open ended question was used to include any other factors they thought relevant to their hypothetical decision making.

**Results:**

Of the 102 patients surveyed, 95 patients (93 %) indicated that they preferred planned over emergency surgery. The most important influencing factor was the presence of specialist supervision (mean rating 9.4, mean rank 1.3) followed by avoidance of operative cancellation (mean rating 8.8, mean rank 2.3) and avoidance of after hours operations (mean rating 8.1, mean rank 3.2). A lower importance was attached to operative timeliness and avoiding prolonged fasting, with reduction in length of hospital stay being the least important variable. There was a direct correlation between absolute ratings and relative rankings independently assigned by patients to each factor.

**Conclusions:**

Patients with hip fractures prefer planned rather than emergency surgery, the presence of specialist supervision being the most important factor influencing their preference.

## Background

Orthopaedic trauma surgery is going through a period of transition whereby trauma cases are being treated as planned events rather than emergencies [1]. In this instance, emergency surgery is being defined as an operation conducted at the first available opportunity following placement on a shared hospital emergency theatre list that is dictated by clinical urgency and hospital resources. In contrast, planned surgery is being defined as an operation conducted during normal working hours on a determined orthopaedic trauma schedule.

Currently, individual surgeon preference and departmental resources guide decision making regarding the use of emergency or planned operating lists for hip fracture surgery. The decision of operative allocation remains a controversial topic among orthopaedic trauma surgeons.

The traditional approach of placing patients with hip fractures on an emergency operating list has the potential advantage of reducing the operative waiting time [2]. The benefits of early surgery are recognised to improve outcomes in older patients with hip fractures, and evidence suggests that delays of >48 h may contribute to an increase in mortality [3–9]. An early operation also allows earlier management of associated pain from the fracture, a reduced length of hospital stay and a quicker return to function [2]. However, an emergency operating list is limited by the availability of hospital resources, and operative delays are common because of competition for limited operating theatre time [10, 11]. The preoperative waiting time and cancellation rate often remain high because more urgent cases take priority [12] and emergency surgery is commonly associated with prolonged or repeated fasting [13] for patients. In addition, emergency operations outside of working hours are less likely to be supervised by consultants [14, 15].

The use of planned operating lists for patients with hip fractures is an emerging trend in orthopaedic trauma surgery, consistent with recommendations from the National Institute for Health and Care Excellence (NICE) guidelines [16]. This shift has resulted in fewer cancellations and fewer after hours operations, along with increased consultant supervision and a reduction in prolonged fasting times [2, 13–15]. In addition, this has allowed time for better preparation of surgery and stabilisation of medical comorbidities, with mixed evidence demonstrating no significant increase in overall mortality [17–24]. Despite this, patients may have to wait longer for scheduled operations and extra operating lists are required to avoid an increase in preoperative and total length of hospital stay [22–25]. The prolonged bed rest whilst waiting for an operation may also be associated with complications due to immobility such as pain [24], pressure sores [25, 26] or venous thromboembolism [27].

The evaluation of patient preference has been used commonly in multiple medical fields including orthopaedic surgery [28–31] but rarely in the investigation of preference for surgical operating time. When varying treatment options exist favouring different benefits and risks that may be valued differently by patients and surgeons, it becomes critical to incorporate and rely on patient preferences in recommending treatment options [32]. However, at present, individual preferences of the surgeon rather than the patient, as well as departmental resourcing and practices, are being used to guide decision making regarding the use of emergency or planned lists for hip fracture surgery. The evaluation of patient preference involves interpreting the relative value patients associate with the relevant variables—consultant supervision, operative timeliness, surgical cancellation, after hours operation, length of hospital stay and prolonged fasting. In guiding surgical planning decisions, we suggest a shift away from this paternalistic model of clinical decision making to a more informative approach. By prospectively surveying patients with hip fractures at a tertiary hospital, we aim to determine the patient preference for emergency or planned hip fracture surgery and the various determinants of the decision making process.

## Methods

### Survey development

The study survey was developed for patients who had sustained a hip fracture, to explore the hypothetical preference for emergency or planned hip fracture surgery. In this instance, emergency surgery is being defined as an operation conducted at the first available opportunity following placement on a shared hospital emergency theatre list that is dictated by clinical urgency and hospital resources. In contrast, planned surgery is being defined as an operation conducted only during normal working hours (8 am to 5 pm, Monday to Friday) on a routine orthopaedic operating schedule. Any surgical procedures that take place outside these normal working hours are defined as after hours operation.

The survey opened with a standard descriptive narrative in simple English, to inform the patient of the injury and the potential benefits and risks associated with either emergency of planned surgery based on current evidence. For emergency surgery, the advantages discussed with patients were the reduction in surgical waiting times and length of hospital stay resulting from early operative fixation. The disadvantages were the relatively higher risks of operative cancellation, after hours operation, prolonged fasting and unsupervised surgery due to the uncertainty surrounding timing for the operation. For planned surgery, it was explained to patients that the risks of these factors were relatively lower; however, the delay in surgery would be associated with prolonged immobility and an increased length of hospital stay. A direct question was then asked regarding hypothetical preference for emergency or planned hip fracture surgery, where the patient was required to make a decision on their own after being given the necessary information. The individual importance of the various factors that contribute to this decision were subsequently assessed including the following: (1) increase in consultant supervision and a reduction in the risk of (2) operative cancellation, (3) waiting time to surgery, (4) after hours operation, (5) extended length of hospital stay and (6) prolonged or repeated fasting. The value for each factor was initially assessed by using a 10-point visual analogue scale, anchored with 0 % value at one end for least importance and 100 % value at the other end for maximum importance. We then asked patients to separately rank each factor from descending importance from 1 to 6, with rank 1 being the most valued and rank 6 being the least valued factor. An open ended question was used to include any other factors that patients thought relevant to their hypothetical decision making. At the end of the survey, demographic data, fracture classification and patient comorbidities using the Charlson Comorbidity Index [33] were also collected by the primary researcher.

The survey was generated after reviewing the current evidence regarding timing of hip fracture surgery, and the individual factors that seem to influence contemporary surgical decision making. The expert opinions of local orthopaedic trauma surgeons were solicited to refine survey clarity, relevance, comprehensiveness and ease of completion. The survey’s content was validated through pilot testing of five participants, confirming that the content accurately reflected the fundamental aspects of the topic before the survey was finalised.

### Survey administration

In this cross-sectional study, patients admitted to our institution following a hip fracture were screened by the principal researcher for eligibility and invited to participate in the study. This occurred after initial acute pain management and stabilisation of medical comorbidities, during the preoperative stage (generally within 24 h of admission) whilst the patient waited for surgery on the orthopaedic ward. This time point was chosen so that patients had the opportunity for medical review and stabilisation prior to conduction of the survey. All interviews were conducted in person at the bedside by the principal researcher (AA) and lasted approximately 15 min. The patients’ families were often present during the interview, but all questions were directed at the patient without influence of family members. Patients excluded were those who were less than 65 years old, did not have the capacity for informed consent, did not have the language capacity to understand the survey or were being managed non-operatively.

It was emphasised prior to the interview that the survey would not influence current surgical management and patients would be presented only a hypothetical choice of preference. Each interview followed a standard format of initially explaining the study to the patient, providing the information sheet and obtaining written informed consent where the patient chose to participate. The principal investigator then read a standard transcript informing the patient of the background for survey questions, following which he handed out the questionnaire to the patient for completion. The researcher collected information on demographics and relevant medical data through a chart review after the interview had ended.

At this institution, all patients with hip fractures were managed with planned surgery, usually 1 to 3 days after admission. This was explained to patients by the principal researcher after administration of the survey. It was also emphasised to patients that despite the use of scheduled operating lists, hip fracture surgery was still a relative emergency operation with preferred operative fixation within a limited time from injury.

A sample size of 100 patients was chosen based on the ability to perform multivariable analysis.

### Data analysis

Using Microsoft Excel and Stata Analysis and Statistical Software v11, descriptive statistics were produced to analyse data obtained from the study. In this process, scores were assigned to each question and univariate analysis of preference was used to examine frequency, mean values and confidence intervals for each response. This was done for the initial question of preference for surgery, as well as each of the determining factors for preference, including the following: consultant supervision, operative timeliness, surgical cancellation, after hours operating, length of hospital stay and repeated fasting. Using logistic regression analysis, patient gender and age were assessed as potential predictors of preference.

## Results

### Characteristics of participants

**Fig. 1:**
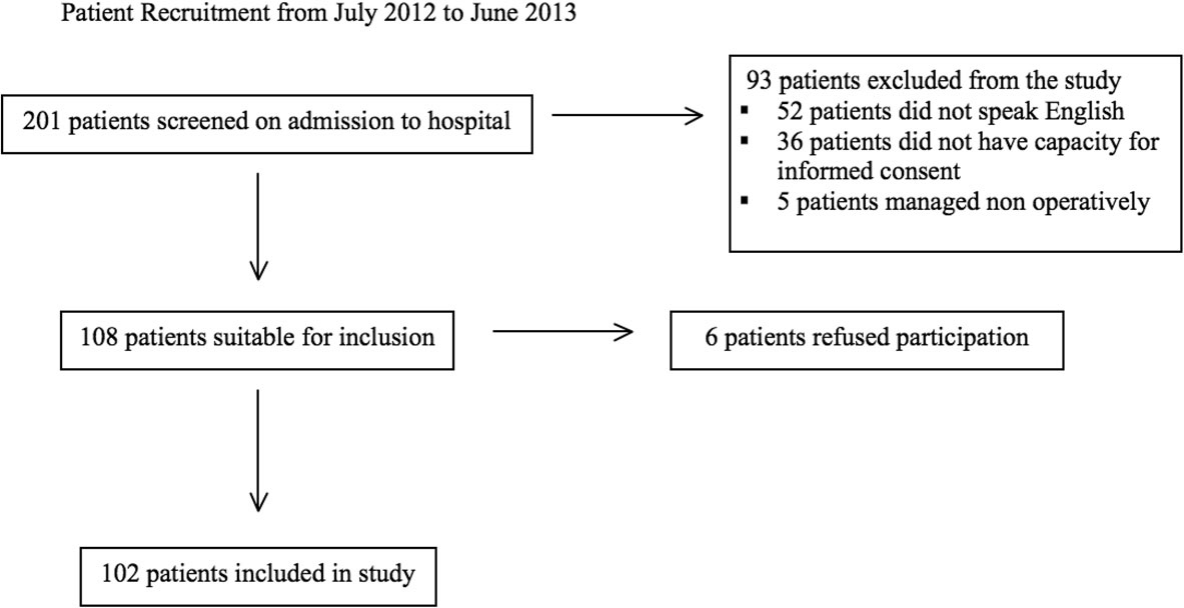
Patient recruitment from July 2012 to June 2013

Between July 2012 and June 2013, we screened 201 patients admitted to a hospital with a hip fracture for potential eligibility. Of these, 52 patients (25.9 %) did not have the language capacity to understand the survey, 36 patients (17.9 %) did not have the capacity for informed consent and 5 patients (2.5 %) were being managed non-operatively. This left 108 patients (53.7 %) who satisfied inclusion criteria, with 102 (50.7 %) consenting for participation in the study as represented in Fig. 1. All participants enrolled completed the questionnaire in its entirety.

Of these 102 patients, 34 (33.4 %) were male and 68 (66.7 %) were female. The mean age of patients was 79.6 years, with the majority (56, 54.9 %) aged between 70 to 84 years. The mean Charlson Comorbidity Index was 2.3 (95 % confidence intervals (95% CI), 1.9–2.8). The complete participant characteristics are summarised in Table 1.

**Table 1:** Patient characteristics (*n* = 102)

		Number	Percent
Gender	Male	34	33.3
	Female	68	66.7
Age (years)	<70	15	14.7
	70–85	56	54.9
	>85	31	30.4
Charlson Comorbidity Index	0–1	46	45.1
	2–4	42	41.2
	>4	14	13.7
Comorbidities	Osteoporosis	22	21.6
	Osteoarthritis	18	17.6
	Smoking (current)	6	5.9
	Overweight (BMI > 25)	6	5.9
Fracture classification	Undisplaced	37	36.3
	Displaced	65	63.7
	Extracapsular	55	53.9
	Intracapsular	47	46.1
Previous hip fractures		11	10.8

Patients with undisplaced (21) or displaced (34) extracapsular and undisplaced (16) intracapsular hip fractures were managed with osteosynthesis using either a dynamic hip screw or cephalomedullary nail. Patients with displaced (31) intracapsular fractures were managed with arthroplasty. This is reflected in Table 2.

**Table 2:** Surgical treatment (*n* = 102)

	Number	Percent
Osteosynthesis	71	69.6
Arthroplasty	31	30.4

### Preferences for emergency or planned surgery

Of the 102 patients surveyed, 95 patients (93.1 %) indicated that they preferred planned hip fracture surgery whilst 7 patients (6.9 %) indicated a preference for emergency surgery (Table 3).

**Table 3:** Patient preference for emergency or planned hip fracture surgery (*n* = 102)

	Patient preference	
	*n*	%
Emergency surgery	7	6.9
Planned surgery	95	93.1
Total	102	100

The importance rating and ranking of each variable is shown in Table 4. There was a direct correlation between absolute rating for the individual factors and the subsequent rank independently assigned by patients to each factor.

**Table 4:** Importance for individual factors that influence patient preference

	Factor	Mean	95 % CI
Rating (0–10)	Increasing consultant supervision	9.4	9.3–9.6
	Reducing risk of cancellation	8.8	8.6–9.0
	Avoiding after-hours surgery	8.1	7.8–8.4
	Reducing time to surgery	7.8	7.4–8.1
	Avoiding repeated fasting	6.8	6.4–7.1
	Reducing length of hospital stay	6.7	6.3–7.1
Ranking (1–6)	Increasing consultant supervision	1.3	1.2–1.5
	Reducing risk of cancellation	2.3	2.2–2.5
	Avoiding after-hours surgery	3.2	3.0–3.4
	Reducing time to surgery	3.7	3.4–3.9
	Avoiding repeated fasting	5.0	4.9–5.2
	Reducing length of hospital stay	5.4	5.2–5.6

Factor rating is from 0 to 10 for absolute importance, where 10 is rated the highest and 0 is rated the lowest. Factor ranking is from 1 to 6 in order of relative importance, where 1 is ranked the highest and 6 is ranked the lowest

In regard to additional factors that influenced preference, 20 patients (19.6 %) volunteered that adequate pain management was important in determining timing for surgery. Patient gender or age did not significantly predict patient preference or value for the individual factors contributing to decision making.

## Discussion

In accepting a patient-centred healthcare model, we should rely on patient preferences to guide clinical decision making involving hip fracture surgery. The results of this study demonstrate that patients with hip fractures overwhelmingly prefer a planned operation (93.1 %) on a scheduled orthopaedic operating list, rather than an emergency operation (6.9 %). This is based on a high perception of value for consultant supervision and reduction in the risks of operative cancellation and after hours operation that are often associated with scheduled operations during normal working hours [2]. Although a scheduled operation may be associated with delays to definitive surgery and an increase in the length of hospital stay, which may produce its own complications due to prolonged immobility [24–27], patients were willing to accept those consequences with lower importance assigned to these determinants of decision making. It is, nevertheless, important to patients that pain is adequately managed preoperatively whilst waiting for surgery. The preference for planned surgery was consistent across gender, age and medical comorbidities in a representative sample of patients with hip fractures.

Although these results are based on a focused population, orthopaedic surgery on the whole is going through a change whereby trauma cases are being treated as planned events rather than emergencies. For hip fractures in particular, the overall evidence and clinical guideline recommendations [1–25] tend to suggest a more balanced decision in the timing of surgery that challenges strict adherence to the traditional emergent approach to surgical management. By following the axiom that individuals are the best judges of their own welfare [34], the key to information exchange lies in evoking patient preference by informing the patient of the benefits and risks associated with each treatment option [35]. The current model of patient-centred care means that we must assess patient preference to align surgical management closer to expectations, and in this instance, it reflects an importance that needs to be placed on providing supervised hip fracture surgery that has reduced risks of cancellation or being performed after hours. The shift towards operating on scheduled orthopaedic trauma lists may bring us closer to achieving these goals.

In the literature to date, there has been little evidence investigating preference for surgical operating time, although patient preference has been used in a variety of surgical fields to assess value for treatment options. Alolabi et al. [28] conducted a recent study of 81 patients at risk for developing femoral neck fractures, using a decision board to assess surgical preference for total hip arthroplasty (THA) or hemiarthroplasty. He found that 75 (93 %) participants chose THA as their preferred operative choice when given the choice. In another study by Gong et al. [30], 78 patients who underwent carpal tunnel release for carpal tunnel syndrome were requested to indicate their preferred level of involvement in clinical decision making, with 59 (76 %) indicating that they preferred a collaborative role. In addition, several studies have emphasised the importance of incorporating patient preferences into orthopaedic care [35–39].

Our study design has several strengths. The survey content was developed based on current evidence and input from local orthopaedic trauma surgeons to inform the variables that may influence patient preference. All data was prospectively collected by a single researcher (AA) and followed a standard survey format, to ensure reliability in data collection. The data is internally validated with the consistency demonstrated between the absolute ratings and the relative ranks for each of the six items that may influence decision making. We used one-on-one interviews to ensure that all participants understood the questions being asked and hence provided their true preferences and had a 94 % response rate for patients eligible for inclusion into the study.

A number of limitations in the study deserve consideration. In sampling a population at a tertiary hospital that manages patients with hip fractures only with planned surgery, there is a possibility of bias towards the treatment modality in the hypothetical scenarios presented to patients. This should not, however, influence the value for the individual factors which contribute to the decision making process. To avoid this risk of bias, the study would need to be conducted at an institution that routinely uses both emergency and planned operating lists for hip fractures. There is further potential for bias given that the questionnaire was administered by what could be perceived by the patient as a prospective surgeon or member of the surgical team and that given responses should please that surgeon. In minimising this impact, a large emphasis was placed on explaining to patients that the survey presented only hypothetical choices and would not influence surgical management. As a cross-sectional study, we conducted all interviews only in the preoperative stage of surgical management with no follow up of patients. In doing so, we did not evaluate the effect that emergency or planned surgery itself would have on influencing patient preference postoperatively. It follows though that the personal operative experience of a patient would create bias either towards or against their preoperative preference and would not contribute to a rational evaluation of preference for surgical operating time, but rather a reflection of their own perceived surgical outcome. Due to the inability to capture every patient prior to operative management, with the principal researcher not being available on site at all times, patients were not consecutively sampled for eligibility and this may have created a potential screening bias. Although we presented patients with information using a transcript based on current evidence prior to the survey, there may be a possible source of information bias based on our chosen wording. However, we attempted to keep the information presented as balanced and objective for both treatment modalities and easy to understand for patients. This study was conducted at a trauma centre in Australia, and our findings may not be applicable to other hospitals with varying departmental resourcing where the choice between emergency or planned hip fracture surgery is not available. This was an observational study, and future studies are required to determine whether patient preference or value for individual determinants of this preference actually impacts on surgical outcomes.

## Conclusions

The majority of patients with hip fractures prefer planned rather than emergency surgery. The increased presence of specialist supervision is valued as the most important influencing factor in decision making. We have provided a patient perspective on various factors concerning the surgical management of hip fractures, and we suggest that this perspective be used to guide planning decisions involving hip fracture surgery in an older patient population.

## Abbreviation

THA: Total hip arthroplasty
